# Artifact segmentation using the U-Net architecture for powder X-ray diffraction images

**DOI:** 10.1107/S160057752600754X

**Published:** 2026-08-25

**Authors:** Albert Vong, Howard Yanxon, Eric Roberts, Hannah Parraga, James Weng, Wenqian Xu, Uta Ruett, Alexander Hexemer, Petrus H. Zwart, Nicholas Schwarz

**Affiliations:** a Argonne National Laboratory 9700 South Cass Avenue Lemont IL60439 USA; b Lawrence Berkeley National Laboratory Berkeley CA94720 USA; SLAC National Accelerator Laboratory, USA

**Keywords:** artifact segmentation, computer vision, machine learning, X-ray imaging

## Abstract

Modern synchrotron X-ray facilities generate X-ray diffraction (XRD) image data at rates that far exceed the capacity of manual analysis, yet critical processing steps such as artifact removal rely on human intervention. Rule-based algorithms in standard beamline software cannot reliably distinguish harmful artifacts like single-crystal diffraction spots from desirable features like preferred orientation, which leads to incorrectly processed data. We demonstrate that a U-Net convolutional neural network trained on expert-labeled experimental data can help automate artifact segmentation in time-resolved XRD measurements of battery materials. To address overfitting from redundant time-series frames, we introduce a mutual information-based pruning algorithm that selects maximally diverse training images. Our optimized model achieves an 85.1% true positive rate on a fully held-out battery dataset while reducing false positives by 34% compared with *GSAS-II*. Critically, the model preserves preferred orientation features that *GSAS-II* incorrectly removes, preventing systematic underestimation of phase intensities in downstream analysis.

## Abstract 2

The capabilities of a U-Net convolutional neural network for masking artifacts in time-resolved X-ray diffraction measurements of battery materials are demonstrated.

## 1. Introduction

Synchrotron X-ray facilities produce massive data volumes using high-brilliance radiation that delivers tens of thousands of times more photon flux than conventional sources (Balerna & Mobilio, 2015 ▸; Winick & Doniach, 2012 ▸). Experiments often involve complex samples with multiple materials and phase components, making data interpretation challenging. Distinguishing relevant data from systematic errors typically requires human expertise due to experiment-specific context, creating a critical bottleneck in analysis pipelines. Automating these human-dependent processing steps is therefore essential to match analysis throughput with the unprecedented data generation rates of modern synchrotron facilities.

X-ray diffraction (XRD) is a non-destructive characterization technique that extracts structural and compositional properties such as grain size, strain and phase composition. It is a crucial tool in research areas such as pharmaceuticals and microelectronics, and is widely performed at synchrotron facilities. During measurement, incoming X-rays diffract off the sample at varying incidence angles and are collected by a 2D detector. At synchrotron facilities with high photon flux, large 2D detectors enable *in situ* and operando experiments that detect subtle events such as structural changes and phase transformations. While 2D images are typically azimuthally integrated to produce conventional 1D XRD patterns for analysis methods like Rietveld refinement, this integration discards azimuthal-dependent information such as preferred orientation that can aid interpretation.

A crucial step in the XRD image processing pipeline is the removal of artifacts – undesired pixel regions including detector artifacts such as saturated pixels and sample-related signals like single-crystal diffraction spots. Upon integration, these artifacts manifest as peaks indistinguishable from real sample signal, potentially causing misidentification of contributing phases in decomposition methods. Existing state-of-the-art beamline software such as *GSAS-II* (Toby & Von Dreele, 2013 ▸) employs segmentation algorithms that automatically identify artifacts using rule-based intensity filtering. However, these algorithms often struggle to differentiate harmful artifacts from desirable but atypical features. For instance, preferred orientation, which is generally a desirable signal, appears as non-uniform intensity distributions along diffraction rings; these are smoother than the localized bright spots characteristic of single-crystal diffraction, yet both produce elevated intensities that are flagged by current software for removal. Consequently, comprehensive artifact removal often requires human intervention to distinguish genuine signal from contamination, as accidental removal of real features is equally detrimental to subsequent analysis.

An alternative to rule-based methods is to model human judgment using neural networks, flexible learned functions trained on labeled examples. By training on datasets where artifacts are labeled while desirable features like preferred orientation are preserved, neural networks can learn nuanced distinctions that rule-based thresholds cannot capture. Neural networks have demonstrated robust performance on X-ray data, including phase identification and quantitative phase analysis (Lee *et al.*, 2020 ▸; Wang *et al.*, 2020 ▸; Szymanski *et al.*, 2024 ▸). However, most XRD-focused neural networks operate on 1D integrated patterns rather than 2D images, positioning them downstream of artifact removal. Like conventional analysis methods, these networks assume artifact-free inputs, typically accounting only for background noise and intensity variations rather than the presence of uncorrelated artifact peaks. Without robust artifact removal, any downstream method inherits contaminated data that compromise reliability. The lack of effective automated artifact masking necessitates human intervention and limits the scalability of autonomous synchrotron XRD experiments, such as self-driving laboratories (Tom *et al.*, 2024 ▸).

In this paper, we demonstrate the capabilities of a U-Net convolutional neural network (CNN) for masking artifacts in time-resolved XRD measurements of battery materials. By training on a curated dataset of human-labeled masks, we show that CNNs can learn artifact identification in the presence of complex features such as shifting diffraction rings, texture and single-crystal spots. We address the challenge of model overfitting on time-dependent data using a mutual information-based filtering method that identifies informative time points for training. Testing on unseen data demonstrates strong performance in the small-data regime, achieving high artifact recall rates.

## 2. Related work

CNNs are deep learning architectures designed for image processing tasks. Their learned convolutional kernels combine features across multiple length scales, using contextual information from neighboring pixels to perform image segmentation. This flexibility enables CNNs to model complex segmentation requirements that challenge rule-based methods, and they have demonstrated success in diverse applications including crack detection (Liu *et al.*, 2019 ▸), urban planning (Abderrahim *et al.*, 2020 ▸) and artifact reduction (Hegazy *et al.*, 2019 ▸).

A key challenge in applying CNNs is the volume and quality of training data required for gradient-based optimization and generalization. Data augmentation techniques can mitigate overfitting when training data are limited, but cannot fully compensate for insufficient sample sizes (Bardis *et al.*, 2020 ▸; Huang & Nowack, 2020 ▸; Nofallah *et al.*, 2022 ▸; Çiçek *et al.*, 2016 ▸). In X-ray machine learning applications, training data are rarely curated from expert-labeled experimental datasets. Instead, most approaches simulate diffraction patterns from pristine crystal structures and apply perturbation strategies to approximate real data. While this enables the generation of large training sets with complete ground truth, essential for quantification tasks like phase identification, it introduces a well documented ‘generalization gap’ between simulated training and real experimental performance (Oviedo *et al.*, 2019 ▸; Belthangady & Royer, 2019 ▸).

Our segmentation task for measurement artifacts, however, does not require quantitative ground truth unlike phase identification, making manually labeled experimental data both feasible and advantageous. Experimental images inherently capture measurement uncertainties, noise and material imperfections that are difficult to simulate accurately. By training on expert-labeled real data, the network learns which features to prioritize under actual experimental conditions, avoiding the transfer learning challenge between simulated and real data. This approach has proven effective for X-ray- specific tasks such as Laue diffraction spot identification (Kirstein *et al.*, 2023 ▸), precipitate and pore identification (Gaudez *et al.*, 2022 ▸), and anomaly classification (Czyzewski *et al.*, 2021 ▸).

The limited data volumes inherent to manual labeling, however, require careful consideration of model usage. Models trained on smaller, material-specific datasets do not generalize to diffraction patterns with different characteristics, which limits effectiveness on new datasets and is a well known problem for neural networks. Nevertheless, for beamlines which specialize in repeated measurements of specific material classes, specialized models can dramatically reduce processing time by automating artifact segmentation that would require extensive human input. This trade-off between domain specificity and automation efficiency makes the approach particularly valuable for high-throughput facilities conducting repeated measurements on similar material systems.

For segmentation tasks, performance metrics include recall (true positive rate), specificity (true negative rate), and balanced metrics such as F1 and DICE scores that penalize both false positives and false negatives. For X-ray artifact classification, however, recall is the critical metric due to extreme class imbalance and the asymmetric impact of classification errors (Saito & Rehmsmeier, 2015 ▸). Artifacts typically constitute less than 1% of pixels, yet upon azimuthal integration even a small artifact can disproportionately impact the integrated intensity at its corresponding 2θ value, potentially causing misidentification in downstream phase analysis (Ashiotis *et al.*, 2015 ▸; McCusker *et al.*, 1999 ▸). Conversely, false positives, *i.e.* incorrectly flagged normal pixels, have minimal impact on integrated patterns since signal at each 2θ is contributed from the entire diffraction ring which consists of thousands of pixels (Ida, 2016 ▸). This trade-off strongly favors maximizing recall, even at the expense of higher false positive rates, to ensure artifact removal before integration.

## 3. Methods

### 3.1. Dataset

**Figure 1:**
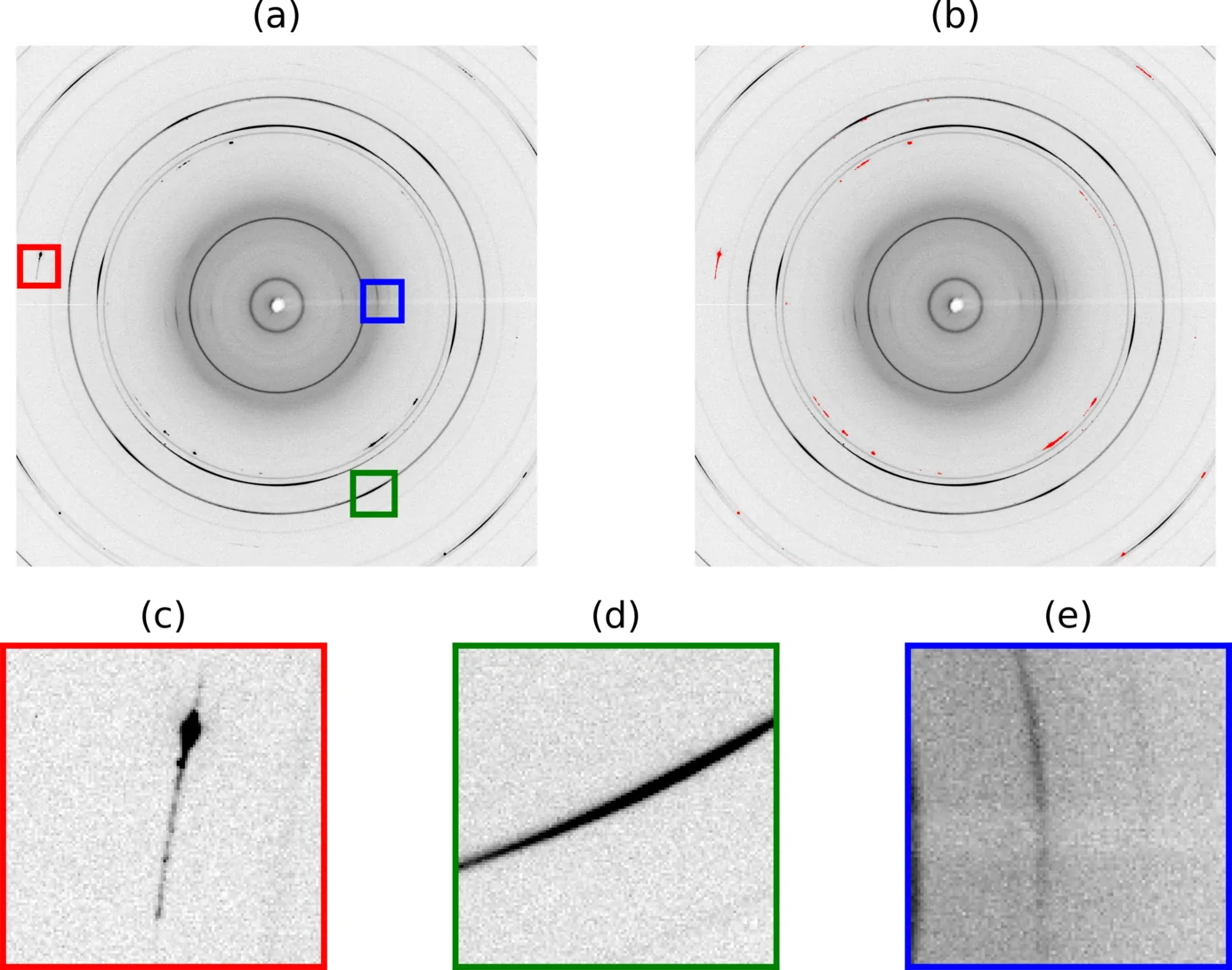
(*a*) An experimental XRD image and (*b*) its masking result. The red, green and blue boxes in (*a*) show (*c*) single-crystal diffraction spots, (*d*) preferred orientation and (*e*) two texture lines.

In this study, we train a battery-specific segmentation model on various datasets of XRD images obtained from *in situ* experiments on battery materials, each containing different types of artifacts (refer to Table 1 ▸). These images are high-resolution 2880 × 2880 pixel intensity arrays captured using a Varex XRD 4343CT area detector. Each XRD image exhibits different characteristics, illustrated in Fig. 1 ▸, due to factors such as photon flux, sample scattering power, effective sample volume and measurement settings. The Nickel83 dataset was collected with a varied temperature profile. The Battery-1, Battery-2, Battery-3, Battery-4 and Battery-5 datasets were collected over the course of battery charging/discharging experiments. For charging/discharging datasets, emerging signals from charge carriers appear as single-crystal spots and textures. All datasets have idiosyncrasies present in time-dependent measurements; consecutively captured ‘snapshots’ of the sample can be highly similar or dissimilar depending on sample dynamics. The consequences of this dataset property are explored in later sections.

The intensities of preferred orientation and single-crystal diffraction spots are often similar in magnitude, making them difficult to separate. Preferred orientation, which is a desirable signal, exhibits differential intensities around a powder diffraction ring, which are typically center-symmetric, which leads to pairs of intense bands separated by 180°. In contrast, single-crystal diffraction spots, which are undesirable, exhibit small and discrete intense spots arising from large crystals. These crystals could either be from the original sample or are formed from *in situ* reactions during the experiment.

**Figure 2:**
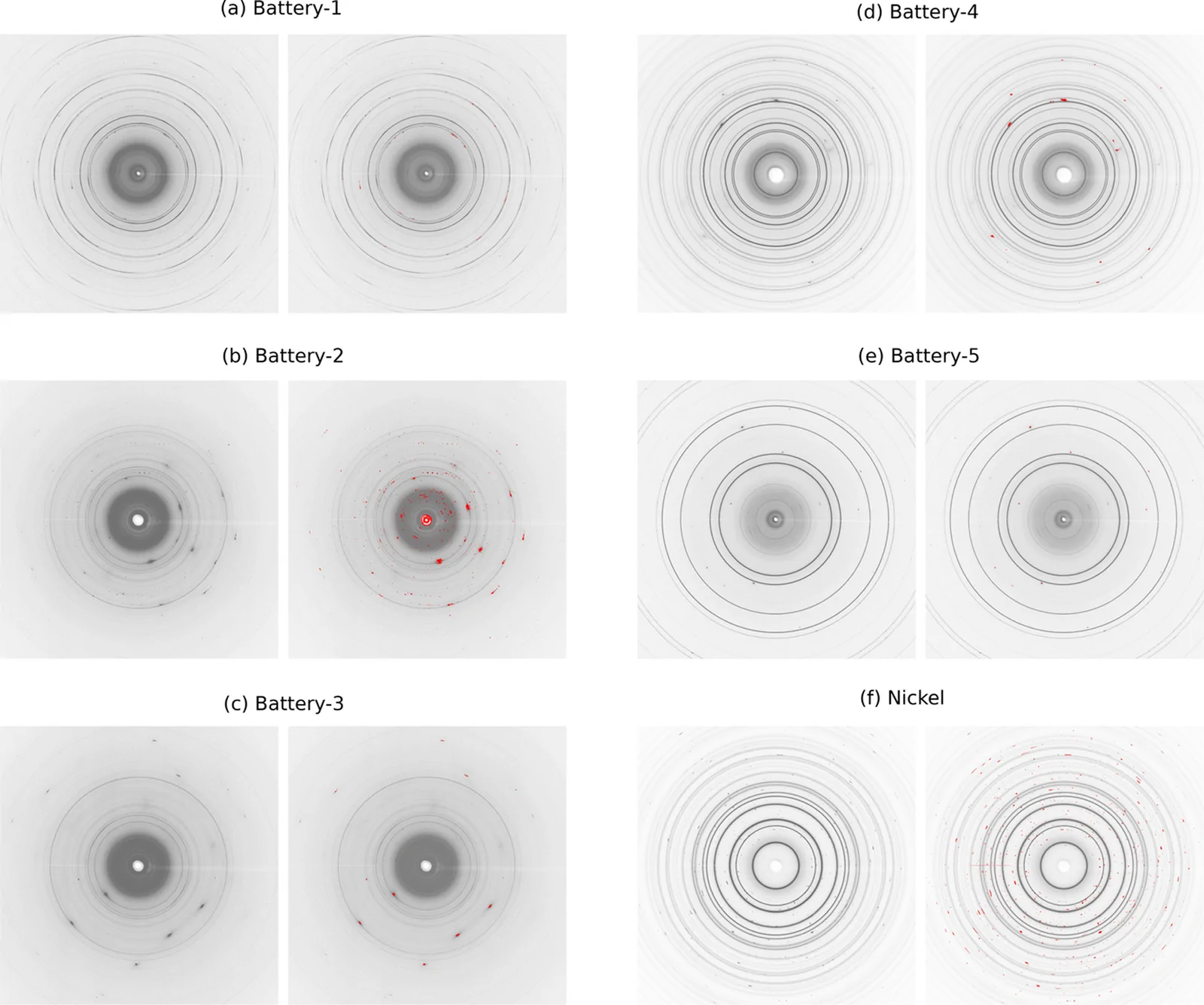
Representative examples from training datasets and the held-out evaluation dataset Battery-5, accompanied by corresponding labels which were generated by combining automated masking with human-curated labeling.

Training labels for these datasets were created in a three-step process. First, automatic artifact labels were generated using a single pixel median outlier algorithm described by Yanxon *et al.* (2023 ▸). Second, images were manually curated, ensuring all single-crystal diffraction spots were accounted for, while removing preferred orientations where necessary. Manual intervention was performed by an experienced beamline scientist, who manually flagged missed artifacts while removing false positives. Third, we masked small detector artifacts by removing masked features less than 5 pixels in size (*i.e.* the number of connected masked pixels is less than 5). This threshold was based on domain knowledge that single-crystal diffraction spots are seldom this small. This allows the CNN to focus on its strength of recognizing larger features rather than few-pixel classification, and the trade-off of excluding this step is explored in Section 4.2. Representative examples of datasets and associated masks are shown in Fig. 2 ▸.

### 3.2. Models and algorithms

#### 3.2.1. Model

**Figure 3:**
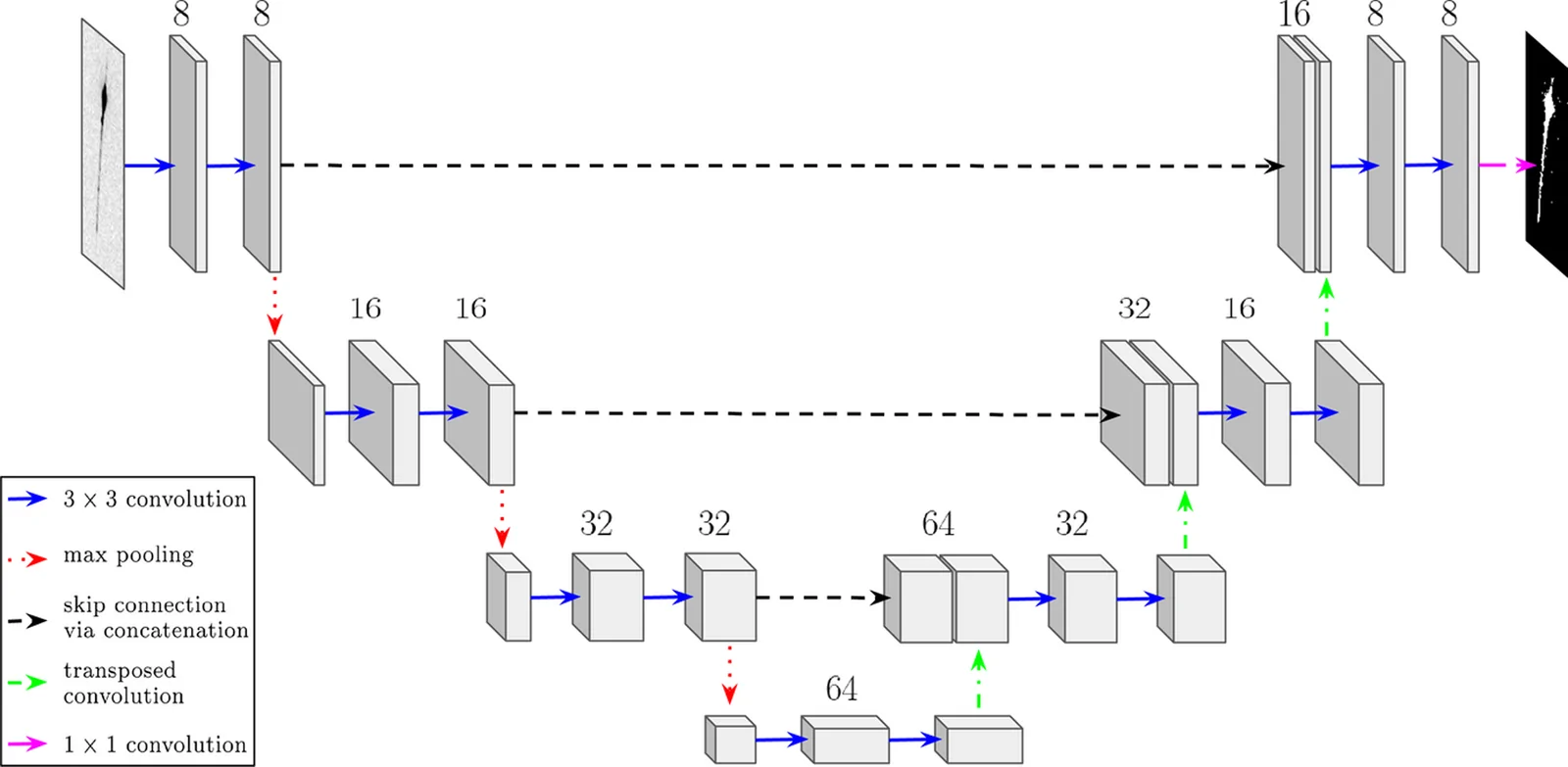
Schematic of a 4-layer U-Net with 8 initial base channels and a convolutional channel growth/decay rate of 2 between encoder/decoder layers. Pictured (left) is a typical grayscale input XRD image after cropping and (right) is the corresponding binary segmentation prediction mask output by the network.

U-Nets are a widely used and highly effective deep CNN originally developed for biomedical image segmentation (Ronneberger *et al.*, 2015 ▸), consisting of a mirrored sequence of contractive and expansive operations at each predefined layer, forming a ‘U’ shape. This specific shape allows the network to aggregate multi-scale feature representations, forming a rich set of learned features which may help with identifying artifacts. A custom U-Net model was implemented using the scientific software package *DLSIA (Deep Learning for Scientific Image Analysis)* (Roberts *et al.*, 2023 ▸), whose API can produce diverse U-Nets, *i.e.* variable depth, growth rate and base channel number. Fig. 3 ▸ shows a U-Net diagram representing one of the many tested in this study, with *d* = 4 layers, *c*_b_ = 8 base channels and a growth rate of *r* = 2. Hyperparameter studies were conducted to optimize the model architecture and are further detailed in the supporting information.

X-ray images are cropped into square windows of varying pixel lengths: 128 × 128, 256 × 256, 512 × 512 and 1024 × 1024. Smaller windows allow the neural net to better identify small single-crystal artifacts, at the cost of losing global context of surrounding preferred orientation pixels. On the other hand, larger windows have the opposite effect, incorporating more image context while reducing individual feature clarity. Therefore, the choice of window size affects the model’s ability to identify artifacts at certain length scales, and this trade-off is explored in Section 3.3. Windows are overlapped, increasing the amount of original training. Cropping and image reassembly is performed using the *qlty* Python package (Zwart, 2021 ▸; Zwart, 2024 ▸).

For training, cropped sub-images are sorted using the mean number of positively labeled pixels, *i.e.* artifacts, prioritizing the sub-images with the most artifacts to combat data imbalance. This is because the majority of pixels are not labeled artifacts, which results in data bias if no sorting step is included. Subsequently, 80% of the cropped images are used to train with 20% withheld for validation, which allows us to explore model uncertainty by sub-sampling the training set.

#### 3.2.2. Data augmentation and normalization

In order to further improve the U-Net prediction, we incorporate additional XRD images, *i.e.* image augmentation, rotating the original images by 90°, 180°, 270° and flipping them along both *x* and *y* axes. No other image manipulation, such as resizing, is done as it may produce unrealistic images, such as non-concentric powder rings. This process increases the number of training sub-images by a factor of 16, so the corresponding model trained with augmented data will be referred to as the 16× model. We also separately incorporate a dataset with perfect powder rings, which we denote as the ‘Perfect’ dataset. The Perfect dataset does not contain any positive labels, which could help reduce false positives associated with preferred orientation lines similar to powder rings.

To normalize between different intensity scales between datasets, an adaptive normalization algorithm was used (see Algorithm 1). This algorithm re-distributes the intensity distribution to highlight any non-smooth features, which likely arise from undesirable artifacts. The *rescale_intensity* and *histogram_equalize* functions from the Python library *sklearn* were used, as referenced in Algorithm 1:
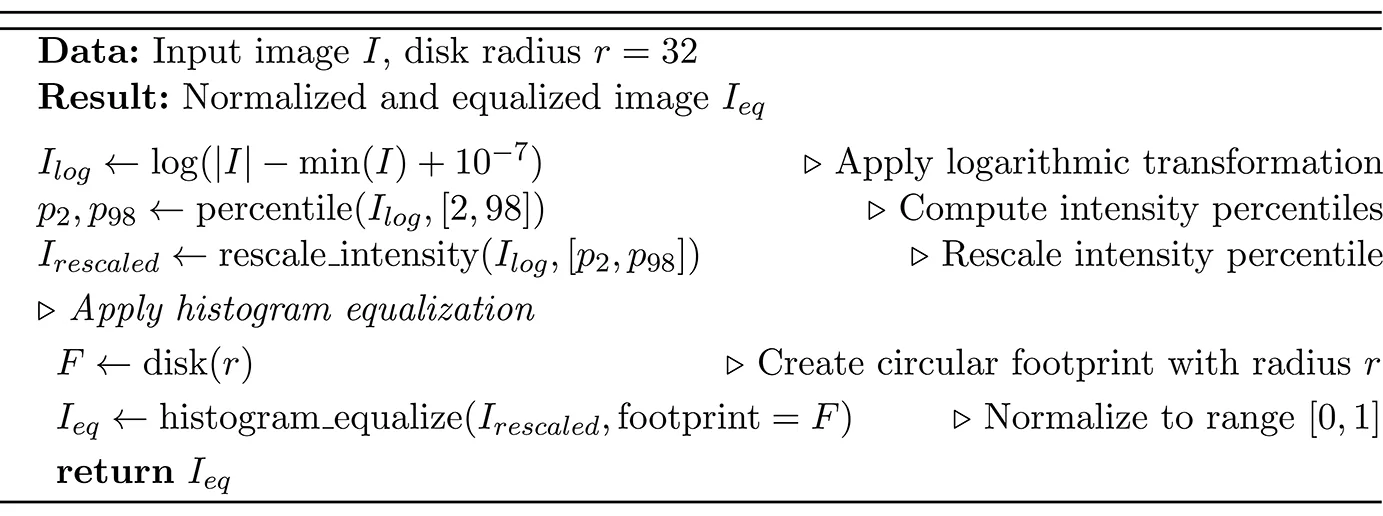


#### 3.2.3. Mutual information pruning

Mutual information (MI) is a distance metric that quantitatively describes the shared information between two probability distributions, and can also be applied to images; the greater the MI, the higher the similarity (Russakoff *et al.*, 2004 ▸). Our *in situ* datasets, which may contain repetitive frames from time-dependent experiments, can therefore be corrected by removing highly similar images. We implement an iterative MI pruning algorithm which selects relatively unique images for training, excluding duplicates which would introduce training bias (see Algorithm 2). We initially calculate the pairwise mutual information of all images in the dataset. We then iteratively select unique images, by first identifying the image with the lowest total pairwise MI, and then removing other images that have similar MI vectors from the dataset. We iterate until no images remain in the dataset.
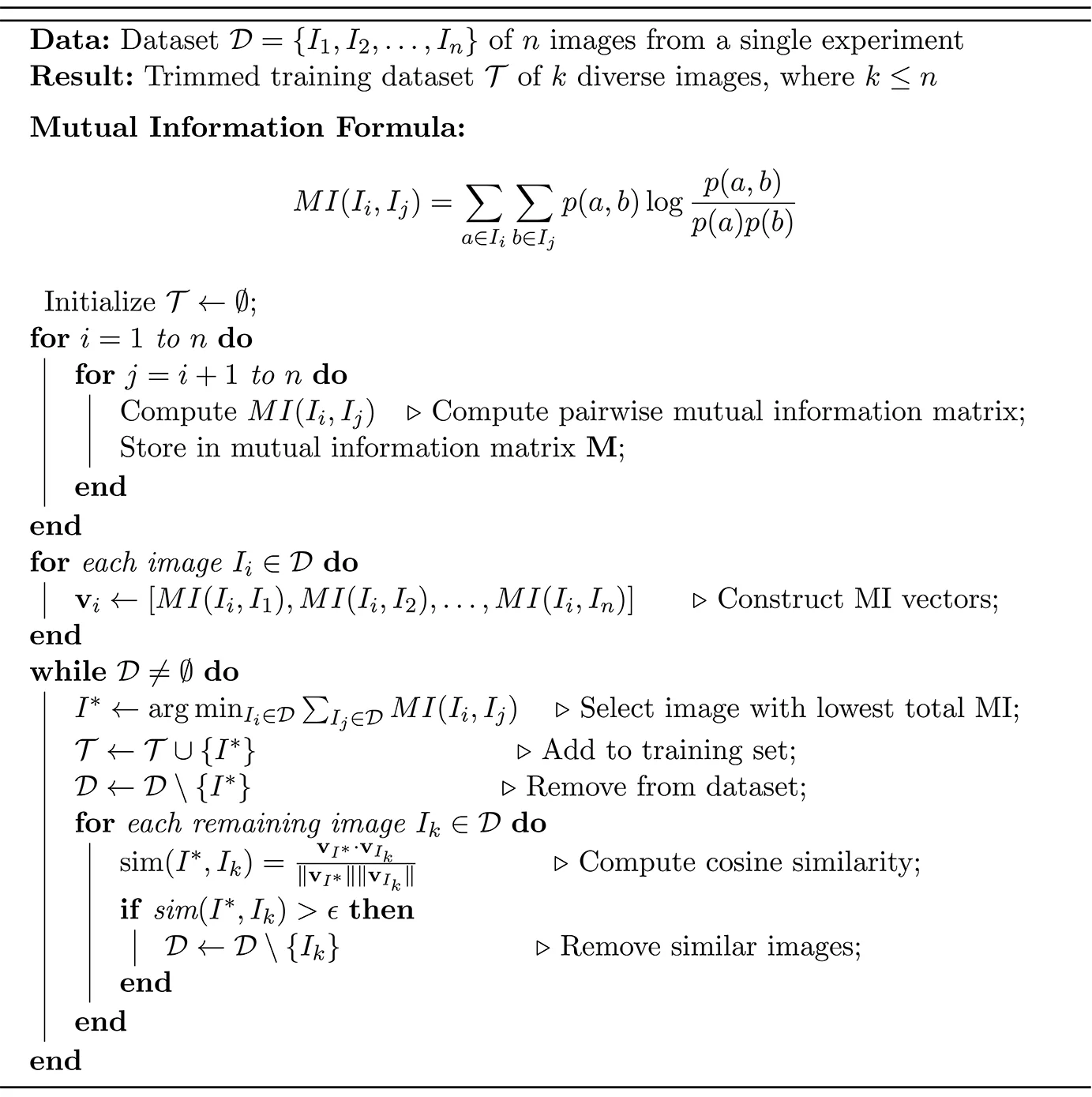


MI pruning is critical when training on images derived from time-dependent processes, since sample changes with respect to time can be gradual or abrupt. This prevents overfitting, since many highly similar images from a specific ‘regime’ may be overrepresented in a dataset due to idiosyncrasies in the experiment itself; for example, if a phase change occurs during the last 20% of an experiment and powder patterns are measured at fixed time intervals, images from the first 80% of an experiment will be largely similar and any machine learning model will learn to overfit on specific patterns captured during this interval. MI pruning would assign equal representation for both phases by only selecting one image from each phase for training. If a random train-validation split was applied on the acquired data without pruning, it is highly likely that similar images would be trained on, highly biasing the model. We will later show the effects of MI pruning on test set accuracy, and how skipping the pruning step harms the model’s ability to generalize.

### 3.3. Training and evaluation details

Raw images from each dataset first go through MI pruning and normalization (see above). This results in 1–4 unique selected images from each dataset. The pruned images not used for training are used as the validation dataset. We crop the processed images into varying window sizes and sub-sample this larger set of cropped images for training with a per-epoch batch size of 50. Two additional unseen datasets, Battery-5 and Z25, are withheld as test datasets to evaluate the model’s ability to predict out of distribution (see Section 4.3). Z25 has specific preferred orientation patterns that *GSAS-II* falsely flags as artifacts, which our model should avoid identifying if trained properly.

The cross-entropy loss function was chosen to score the network predictions against the ground truth and was optimized using the ADAM optimizer (Kingma & Ba, 2014 ▸) to update the U-Net weights during training. The learning rate was set to a static 10^−2^ and all training was performed on an Nvidia A100 GPU with 80 GB of memory. As previously discussed, recall was used as an evaluation metric for training, validation and test datasets. To minimize the effects of randomization on other aspects of training, a constant fixed seed was used to instantiate model parameters regardless of model size. A separate seed was used to randomize the order of images during training, which affects the order and selection of images used in training.

## 4. Results

In this section, we examine the impact of pruning, incorporating additional data biases, and image augmentation on performance. We also evaluate out-of-distribution model performance on our test datasets, and demonstrate the downstream impact of correctly labeling preferred orientations with our model, versus the conventional baseline *GSAS-II* algorithm. For all experiments in this section, we use an optimized U-Net model whose hyperparameter selection is described in the supporting information.

### 4.1. Image augmentation

We investigate the effects of image augmentation, *i.e.* rotation, reflection and inclusion of the Perfect dataset. The 16× model, which includes augmented data but lacks the Perfect dataset, is first compared against the 1× model whose training dataset is not augmented in any way. Both models are trained on all datasets except Battery-5, which was chosen by a domain expert and masked using the same procedure, but was never examined or optimized against. Strikingly, the 16× (1×) U-Net model with all optimized hyperparameters, pruning and modified masking achieves a true positive rate of 85.1% (80.7%) for the Battery-5 dataset, showing some capability to generalize out-of-distribution that improves when training on augmented images. The average false positive rate of the 16× model is also reduced by 34% and 36% in comparison with the *GSAS-II* algorithm masking and 1× model, respectively, the effects of which are explored in Section 4.3.

We then augment the 16× model with the Perfect dataset, which consists only of perfect concentric powder rings with all negative labels. We observed a small increase of 3% in false positive counts compared with the vanilla augmented dataset and a true positive rate of 84% on the test dataset, which is lower than the 16× model by itself. While surprising, this could be because the addition of many negative labels is not informative for a CNN to better distinguish ambiguous artifacts, such as the distinction between preferred orientation and single-crystal diffraction spots.

### 4.2. MI pruning and additional masking

MI pruning was introduced as a method to prevent image over-representation from time-series images. We repeat the same experiments using the 16× model, but include all images from each material class instead of using a pruned image subset. We observe a drop in test set accuracy when we remove MI pruning, from 85.1% to 78%, showing that sample over-representation will hurt the model’s ability to generalize despite having a much larger training set size; if given the opportunity, the model will memorize the most frequent examples seen during training.

We also check the validity of the pixel island masking step described in Section 3.1. Without this masking step, we keep single or few-pixel features present in the dataset, since the median outlier algorithm evaluates pixels individually and the manual masking does not remove all of these labels. Since CNNs excel at identifying larger features through the receptive fields, trying to segment artifacts at the single pixel level is equivalent to predicting noise, which is incredibly difficult. We observe a large drop in accuracy, shown in Table S2 in the supporting information, likely because we force the model to identify measurement artifacts, *i.e.* noise, on top of single-crystal spots. When analyzing further, 20–30% of auto-labeled features fall in this small-pixel threshold, which explains the large drop in model performance, since the model performs especially poorly on predicting few-pixel detector artifacts. We recommend that a separate model be trained and fine-tuned on detecting detector artifacts, since they are fundamentally different to crystal artifacts which makes it difficult to simultaneously optimize a network for both.

### 4.3. XRD data analysis

To illustrate the impact of artifact masking on downstream analysis, we first use the Battery-1 dataset as a case study. This dataset was acquired during an *in situ* synchrotron powder XRD experiment on a lithium-ion battery cell with known composition. As described in Table 1 ▸, the images contain single-crystal diffraction spots, powder rings with uniform intensity, and rings with non-uniform intensity indicative of preferred orientation, with contributions from NMC (nickel, magnesium, cobalt) cathode material, lithium, aluminium and graphite phases.

**Figure 4:**
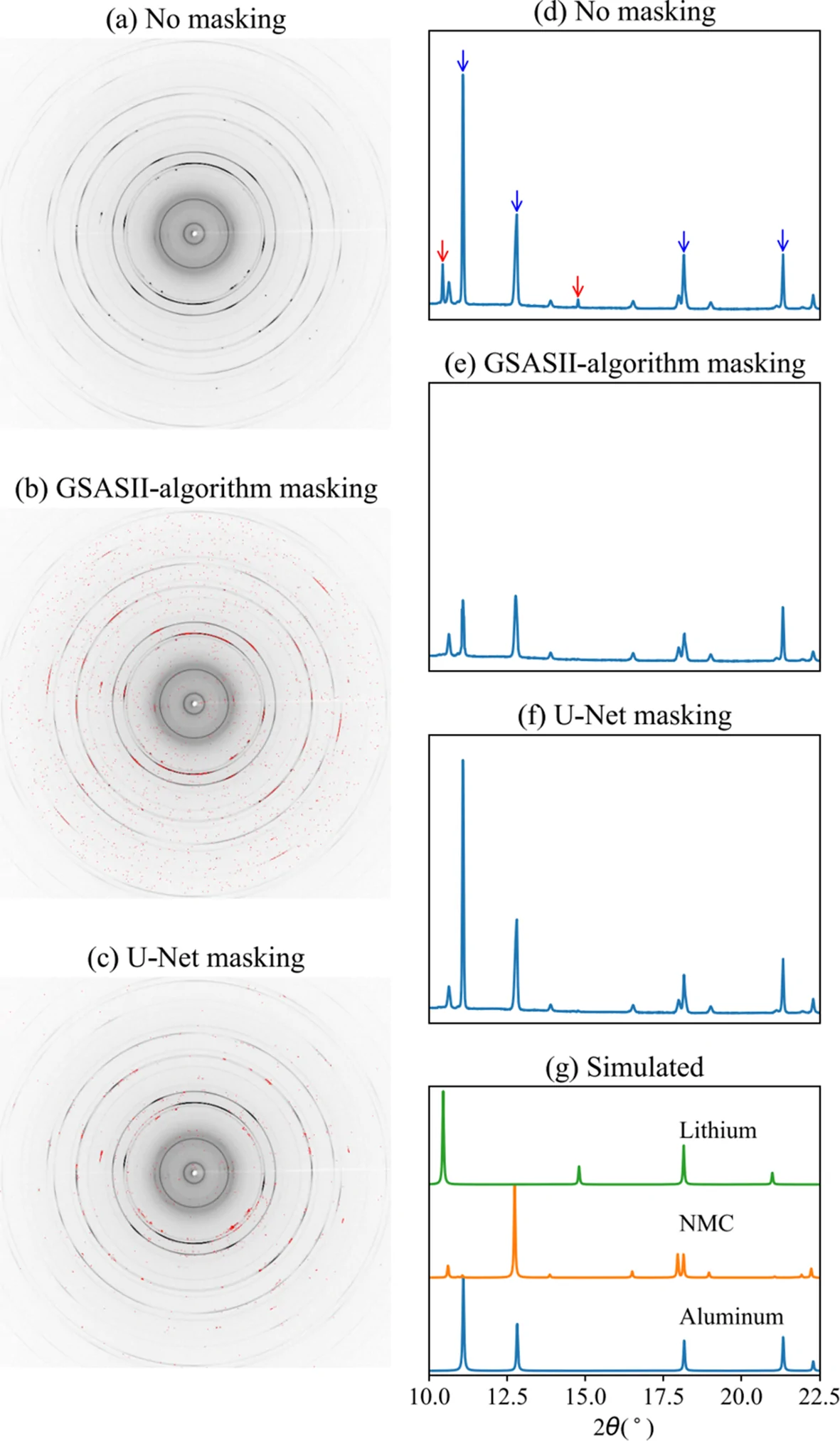
An XRD image of Battery-1 dataset (*a*) without mask, (*b*) with *GSAS-II* algorithm mask and (*c*) with U-Net mask. The corresponding integrated 1D patterns are displayed in (*d*), (*e*) and (*f*), respectively. Panel (*g*) shows the calculated pattern from the three main phases in the sample, NMC, aluminium and lithium. Some peaks from the aluminium and lithium phases are marked by blue and red arrows, respectively, in (*d*).

Fig. 4 ▸ shows a Battery-1 image with (*a*) no mask, (*b*) *GSAS-II* mask and (*c*) U-Net mask, alongside their integrated 1D patterns (*d*), (*e*) and (*f*). Integration converts the 2D image into a 1D intensity versus 2θ pattern by binning pixels sharing the same calibrated 2θ value; masked pixels are excluded from this summation. The lithium diffraction spots (red arrows) and aluminium preferred orientation peaks (blue arrows) are the primary features of interest in this comparison. Both methods successfully remove the lithium single-crystal peaks from the 1D pattern. However, the *GSAS-II* algorithm also masks preferred orientation segments of the aluminium rings (leftmost blue arrow), reducing the integrated intensity of the largest aluminium peak by over 70%. This false positive masking leads to a systematic underestimation of aluminium phase intensities. The U-Net avoids this, producing aluminium peak intensities consistent with the calculated pattern [Fig. 4 ▸(*g*)], which assumes no preferred orientation.

**Figure 5:**
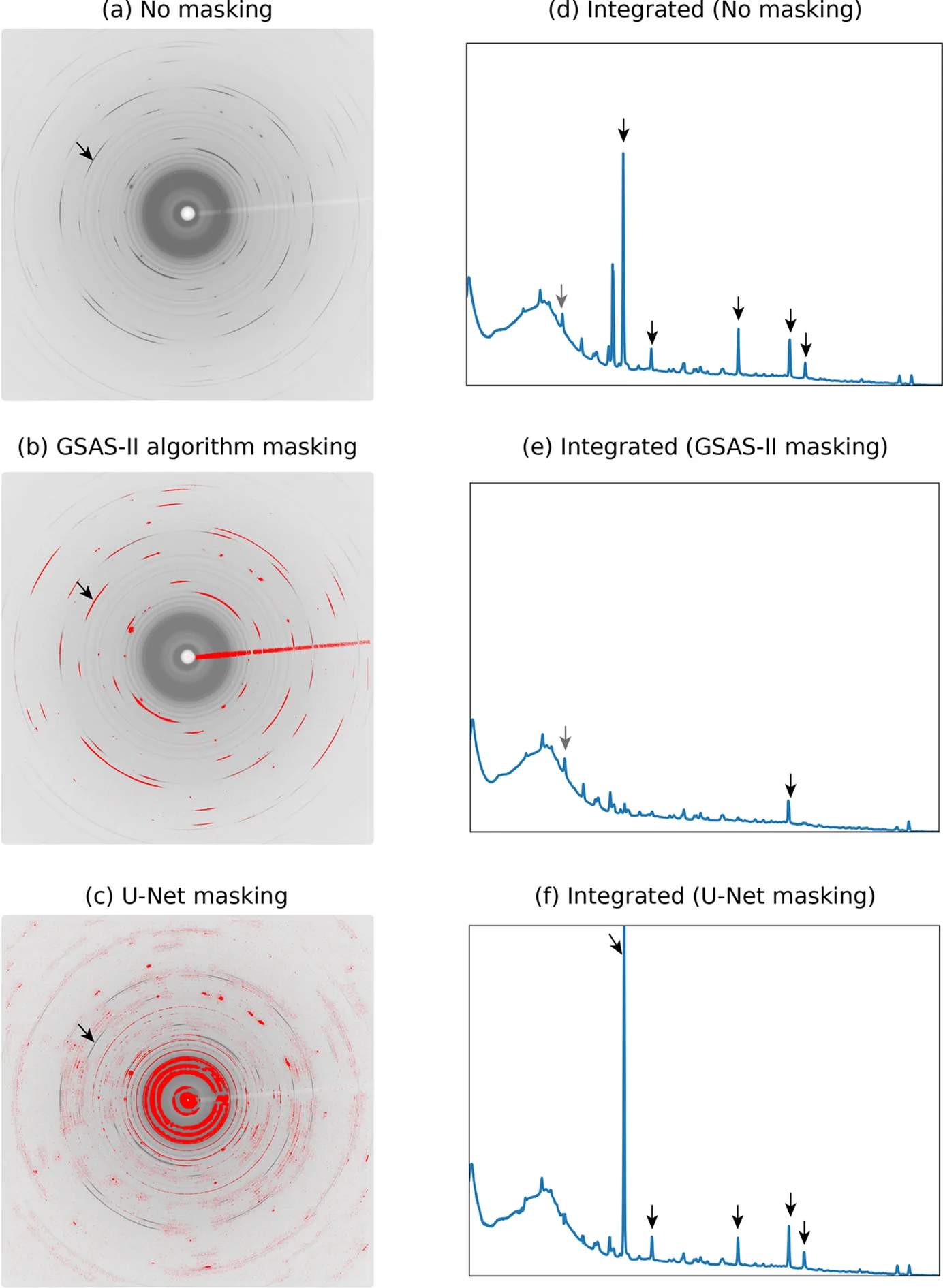
An XRD image of Z25 dataset (*a*) without mask, (*b*) with *GSAS-II* algorithm mask and (*c*) with U-Net mask. There are preferred orientation textures (black arrow, left) that are flagged by *GSAS-II*, which removes them from the integrated spectrum. The U-Net aggressively masks center pixels, but avoids almost all preferred orientation patterns due to its training. The corresponding integrated 1D patterns are displayed in (*d*), (*e*) and (*f*), respectively. Black arrows indicate peaks that are either partially or fully suppressed by *GSAS-II*, but are correctly shown by the U-Net, showing how neural networks can encode human preferences.

We further evaluate the U-Net on Z25, an unseen battery cathode dataset with no known ground truth phases (Fig. 5 ▸). Like Battery-1, Z25 contains single-crystal spots overlapping with strong preferred orientation features. *GSAS-II* masks both, suppressing the intensity of numerous peaks in the integrated spectrum (black arrows). The U-Net correctly preserves preferred orientation features while masking single-crystal spots, yielding a more complete spectrum [Fig. 5 ▸(*f*)]. We do note that the U-Net aggressively masks pixels near the beam center, including several low-2θ diffraction rings. This is likely a consequence of their high relative intensities combined with insufficient negative training examples in this region. The effect on the integrated spectrum is minor – largely absorbed by background signal – with the exception of one peak [gray arrow, Figs. 5 ▸(*d*)–5 ▸(*e*)]. Some additional masking of background pixels adjacent to preferred orientation streaks is also present, but this does not significantly affect peak intensities.

Together, these examples demonstrate that the U-Net more faithfully encodes the masking intent: removing artifact pixels while preserving physically meaningful signal. Residual failure modes, such as over-masking near the beam center, point to the need for more diverse negative training examples. Nevertheless, these results are encouraging for deploying this approach at high-throughput facilities, where measurements are typically domain-specific and sample-repetitive, reducing the risk of out-of-distribution inputs that would normally require additional training data. This would nevertheless require manual labeling and training for a smaller dataset, and would require careful monitoring of measured samples to ensure minimal sample drift.

## 5. Summary and outlook

In summary, we trained a U-Net machine learning model to identify single-crystal artifacts from XRD patterns of battery materials measured at the Advanced Photon Source (APS). By incorporating data pre-processing steps to account for image similarity and detector artifacts, in addition to hand-curating dataset labels to encode human preferences, our model demonstrated some generalization capability; we observed a 85.1% true positive prediction rate for single-crystal artifacts on an unseen battery test dataset that we did not observe or optimize against. Our model also avoids falsely masking preferred orientation versus the baseline *GSAS-II* algorithm, which normally requires a lengthy manual curation step to achieve. Removing falsely flagged artifacts is crucial to prevent phase misidentification in subsequent steps in the typical analysis pipeline.

Several X-ray light source facilities are currently undergoing or have recently undergone next-generation upgrades, such as the recent upgrade of the APS. This has led to a several orders of magnitude increase in data volumes (Schwarz *et al.*, 2020 ▸), making surrogate models such as the U-Net necessary to process large volumes of image data in a timely manner. In the current study, we demonstrated the feasibility of this model on a smaller curated set of experimental data; we plan on exploring model performance on much larger, varied datasets spanning many more material classes for a production-ready model. For example, we would need to design a modified heuristic for calculating mutual information, since pairwise mutual information scales poorly with dataset size. We also stress the importance of designing more specific models to catch certain types of artifacts; instead of training one model as a catch-all method, it may be more feasible to develop a combination of models that specialize in detecting different kinds of features; these can then be used in tandem to selectively mask features of interest. For example, the U-Net developed in this paper is specifically for single-crystal artifacts in *in situ* battery experiments. Future work could explore models for phase identification, although additional work would be needed to generate labeled data at scale. Overall, our approach establishes that neural networks can approximate expert judgment in artifact removal, a promising step towards automating processing pipelines that match the data throughput demands of modern synchrotron facilities.

## 6. Code and reproducibility

Due to the size of the augmented image dataset, the original study was carried out on compute nodes with 512 GB of RAM. To improve model accessibility, a more lightweight implementation has been included alongside the original model and training script on the project GitHub page: https://github.com/AdvancedPhotonSource/AIRXD-CNN-PUB. The most optimized model trained on our computational resources is also included for replicability. In the lightweight implementation, an image augmentation pipeline was used, *i.e.* random augmentation, instead of including all augmented images in the training set, greatly reducing the memory requirements for training data and allowing for training on a local machine. Note that model accuracy is not the exact same as the original implementation due to this change in the training pipeline. An example notebook is included in the repository, showcasing the lightweight implementation.

## Supplementary Material

Supporting information. DOI: 10.1107/S160057752600754X/zhu5011sup1.pdf

## Figures and Tables

**Table 1:** Characteristics of datasets SCD spots and POs stand for single-crystal diffraction spots and preferred orientation, respectively. Note: all images contain uniform rings and detector artifacts.

Dataset	No. of images	Characteristics
Nickel83	11	SCD spots
Battery-1	11	SCD spots and POs
Battery-2	12	SCD spots and textures
Battery-3	12	SCD spots and textures
Battery-4	14	SCD spots and textures
Battery-5	12	SCD spots and textures
Perfect	27	Only perfect powder rings
Z25	10	SCD spots and POs
