# Supplementary material for: Artifact segmentation using the U-Net architecture for powder X-ray diffraction images
Source: J Synchrotron Radiat. 2026 Aug 25;33(Pt 5):1522–30. doi: 10.1107/S160057752600754X (PMC13549334; doi:10.1107/S160057752600754X)
Supplement: Supplementary file 1 [file s-33-01522-sup1.pdf]

JOURNAL OF SYNCHROTRON RADIATION

Volume 33 (2026)

Supporting information for article:

Artifact segmentation using the U-Net architecture for powder X-ray diffraction images

Albert Vong, Howard Yanxon, Eric Roberts, Hannah Parraga, James Weng, Wenqian Xu, Uta Ruett, Alexander Hexemer, Petrus H. Zwart and Nicholas Schwarz

Supplemental Information: Artifact Segmentation using the U-Net Architecture for Powder X-ray Diffraction Images

Albert Vong,[a]* Howard Yanxon,[a] Eric Roberts,[b] Hannah Parraga,[a] James Weng,[a] Wenqian Xu,[a] Uta Ruett,[a] Alexander Hexemer,[b] Petrus H. Zwart[b] and Nicholas Schwarz[a]

[a]*Argonne National Laboratory, 9700 South Cass Avenue, Lemont, IL 60439 USA, and* [b]*Lawrence Berkeley National Laboratory. E-mail: avong@anl.gov*

1. Training Details

1.1. U-Net Optimization

The U-Net was optimized by varying training epochs and the depth of the U-Net architecture, separately. The dataset preparation is described in the main text and remains the same for all training runs. For replicability, each training hyperparameter set is repeated 5 times. Training is conducted using a fixed $c_b = 8$ and a growth rate of 2. Hence, the number of learnable parameters for the U-Net varies based on the depth, with 6,562, 29,650, 121,394, and 487,154 parameters for depths 2, 3, 4, and 5, respectively. All model metrics are calculated only from the validation set, which was withheld from training.

We examine the impact of U-Net depths and window sizes on true positive rates using the Nickel83, Battery-1, Battery-2, Battery-3, and Battery-4 datasets. The U-Net architectures were trained for up to 100 epochs with window sizes of of 128×128,

256×256, 512×512, and 1024×1024 as previously discussed. Figure 1 illustrates that the true positive rate is maximized at depth = 3 at all window sizes. The Battery1 dataset generally performs worse than the others as it has the most artifact diversity, as previously explained. Decreased overall performance at larger window sizes suggests that identifying single crystal artifacts relies more on their small individual features rather inferring from larger, surrounding patterns. Worse performance at greater depths indicates, given the data set size, a deeper U-Net is over-parametrized for the given problem, and does not have sufficient data to fit. The depth = 3 setting balances accuracy and computational demand, and additional training is conducted based on this configuration.

We use the same datasets to optimize the number of training epochs. The model that is trained using these sub-images will be referred to as the 1X model for future reference. Tables 1 and 2 showcases the true positive rate accuracy of U-Net networks against the validation dataset at 5, 10, 20, 30, 50, 75 and 100 epochs with the optimal U-Net depth of 3. There are several global trends: First, epochs past 50 show a decrease in true positive rate which is a sign of overfitting, especially on the Battery1 dataset. This suggests that the U-Net has learned to identify single-crystal features with our limited dataset size in fairly few iterations. Second, we note that the larger window models perform especially poorly on the varied dataset Battery1, likely due to the heterogeneity of artifacts; a smaller window would allow the model to focus on fewer artifacts at a time to discriminate them.

The 256×256 model balances overfitting versus feature extraction, having comparable accuracies to the smaller 128×128 model but lower standard error. We omit results from the 1024×1024 model despite having evaluated its recall, because the recall is too low to be viable. From this hyperparameter sweep, we choose the U-Net model with a depth of 3 and epoch number of 30 as the optimized hyperparameter

choice for additional model evaluation.

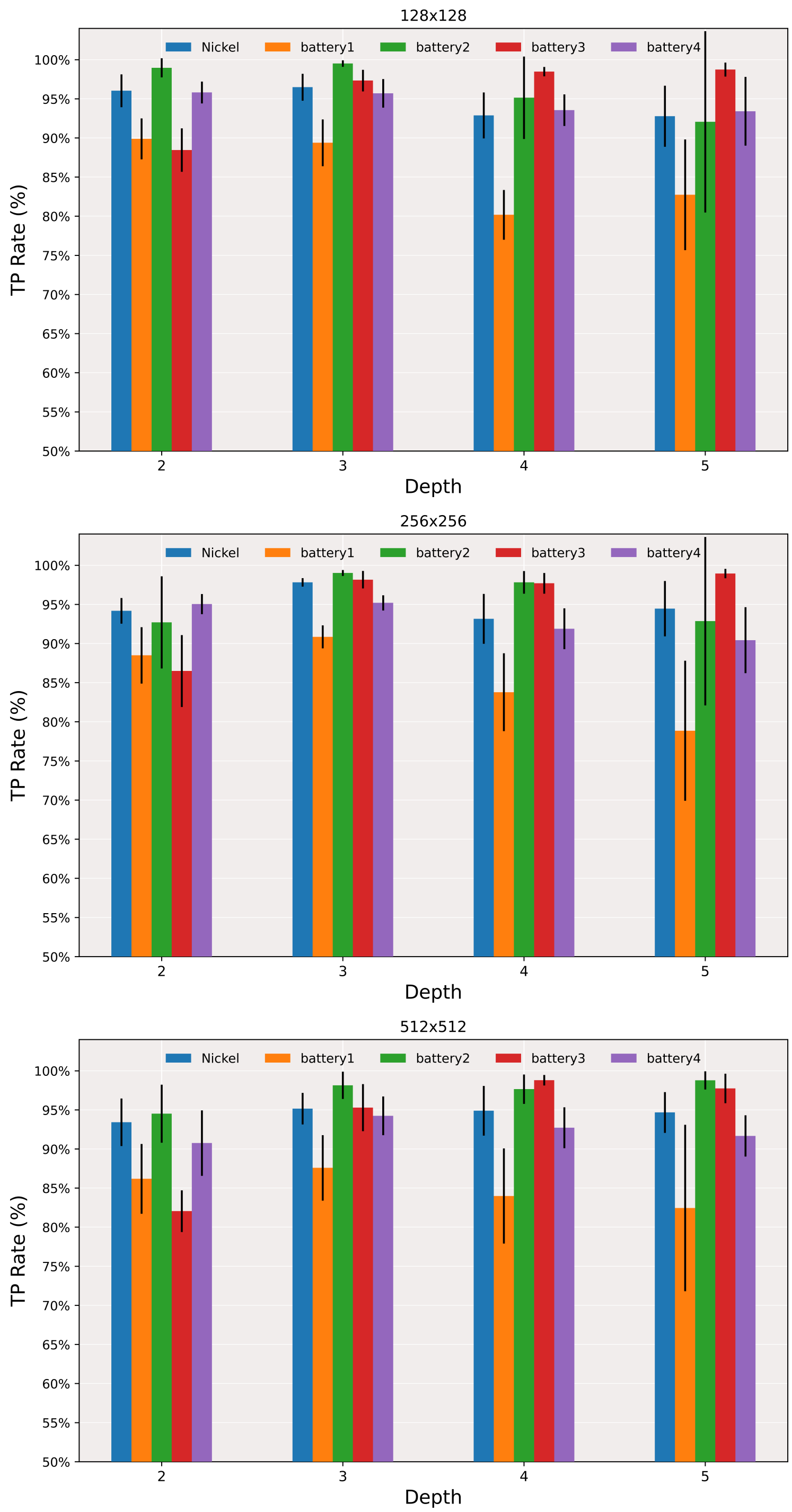

Fig. 1. True positive rates on validation set data as a function of U-Net depth of test datasets.

Table 1 (pruned, with masking):

Table 1. *True positive rate on validation set data for depth = 3 model at different epochs with pruning and masking.*

Sub-images	Dataset	Epochs 10	20	30	50	75	100
128×128	Nickel83	94.8 ± 4.0	94.1 ± 3.2	95.7 ± 2.9	92.9 ± 2.9	91.4 ± 3.5	88.9 ± 6.4
	Battery-1	88.3 ± 5.0	88.6 ± 3.9	83.9 ± 3.6	80.2 ± 3.2	79.3 ± 5.4	77.9 ± 4.5
	Battery-2	96.4 ± 6.1	98.5 ± 1.0	99.1 ± 0.8	95.1 ± 5.3	96.7 ± 2.2	96.7 ± 1.8
	Battery-3	97.2 ± 0.8	98.9 ± 0.3	98.7 ± 0.5	98.5 ± 0.6	98.1 ± 1.0	97.8 ± 0.8
	Battery-4	92.8 ± 1.8	93.7 ± 2.1	94.5 ± 1.9	93.6 ± 2.0	93.7 ± 1.7	92.6 ± 1.1
256×256	Nickel83	93.5 ± 3.2	94.1 ± 3.5	95.1 ± 1.9	93.2 ± 3.2	92.6 ± 3.8	90.3 ± 5.6
	Battery-1	84.9 ± 7.9	87.3 ± 3.7	88.4 ± 1.7	83.8 ± 5.0	80.4 ± 5.0	80.5 ± 5.1
	Battery-2	99.3 ± 0.6	98.8 ± 0.7	97.8 ± 0.9	97.8 ± 1.4	98.3 ± 0.9	96.9 ± 3.2
	Battery-3	97.8 ± 0.9	98.8 ± 0.7	99.3 ± 0.6	97.8 ± 1.4	98.3 ± 0.9	96.9 ± 3.2
	Battery-4	93.0 ± 2.3	94.2 ± 1.0	95.4 ± 1.0	91.9 ± 2.6	92.4 ± 3.7	92.0 ± 3.5
512×512	Nickel83	90.7 ± 9.5	85.7 ± 12.4	92.6 ± 5.9	94.9 ± 3.2	95.4 ± 4.8	92.1 ± 5.7
	Battery-1	81.9 ± 10.3	78.0 ± 12.2	76.7 ± 13.9	84.0 ± 6.1	81.8 ± 11.0	77.4 ± 6.7
	Battery-2	95.9 ± 4.5	88.0 ± 18.3	98.0 ± 2.1	97.7 ± 1.9	98.3 ± 1.9	93.7 ± 9.6
	Battery-3	90.1 ± 5.1	94.0 ± 4.9	96.7 ± 1.6	98.8 ± 0.7	98.3 ± 0.9	98.1 ± 0.6
	Battery-4	88.5 ± 4.0	93.5 ± 1.7	93.9 ± 1.7	92.7 ± 2.6	91.4 ± 2.7	91.4 ± 2.5

Table 2 (pruned, no masking):

Table 2. *True positive rates on validation set data for depth = 3 model with pruning and no masking.*

Sub-images	Dataset	Epochs 10	20	30	50	75	100
128×128	Nickel83	94.2 ± 1.7	92.0 ± 3.3	94.6 ± 1.7	90.9 ± 2.8	92.3 ± 2.5	88.1 ± 6.3
	Battery-1	77.4 ± 4.3	70.4 ± 7.0	76.1 ± 5.1	65.1 ± 8.5	73.8 ± 3.7	64.6 ± 9.2
	Battery-2	92.6 ± 0.6	92.6 ± 0.5	92.3 ± 0.5	88.7 ± 6.0	91.9 ± 0.7	91.5 ± 1.4
	Battery-3	91.0 ± 4.8	95.5 ± 0.5	95.3 ± 2.5	95.3 ± 1.0	93.0 ± 2.2	94.7 ± 1.2
	Battery-4	91.3 ± 3.8	89.4 ± 3.0	91.2 ± 1.2	90.0 ± 2.7	88.9 ± 1.1	89.3 ± 1.8
256×256	Nickel83	85.5 ± 17.1	92.0 ± 3.3	94.6 ± 1.8	90.6 ± 3.0	92.8 ± 2.7	90.2 ± 2.8
	Battery-1	73.2 ± 12.9	71.9 ± 7.7	67.4 ± 5.1	70.9 ± 7.0	66.4 ± 4.1	70.7 ± 3.8
	Battery-2	89.0 ± 7.9	92.2 ± 0.9	92.0 ± 0.5	91.4 ± 1.3	91.9 ± 0.6	90.8 ± 2.8
	Battery-3	87.2 ± 5.7	94.9 ± 2.7	92.9 ± 3.6	95.9 ± 0.8	95.3 ± 2.5	94.7 ± 1.5
	Battery-4	88.3 ± 4.2	90.4 ± 3.0	91.5 ± 1.5	88.2 ± 4.1	88.2 ± 4.8	88.0 ± 1.3
512×512	Nickel83	89.4 ± 7.0	78.8 ± 6.8	90.8 ± 4.1	93.6 ± 3.1	94.9 ± 2.2	95.2 ± 2.7
	Battery-1	67.9 ± 7.4	63.5 ± 5.7	70.8 ± 7.7	77.1 ± 4.2	66.9 ± 5.6	79.0 ± 2.6
	Battery-2	91.0 ± 4.0	86.7 ± 5.2	91.2 ± 1.2	92.8 ± 0.5	92.1 ± 0.6	92.7 ± 0.4
	Battery-3	77.4 ± 7.5	64.8 ± 9.1	87.6 ± 6.2	88.4 ± 4.9	93.3 ± 3.3	95.1 ± 3.7
	Battery-4	87.4 ± 1.9	80.9 ± 5.9	87.8 ± 5.5	89.0 ± 3.2	90.1 ± 3.8	92.6 ± 2.9
